# Characteristic competencies for effective teachers in healthcare education

**DOI:** 10.31744/einstein_journal/2025RW1360

**Published:** 2025-08-12

**Authors:** Marina Iahn-Aun, Marcelo Vivolo Aun, Welbert Oliveira Pereira

**Affiliations:** 1 Hospital Israelita Albert Einstein Faculdade Israelita de Ciências da Saúde Albert Einstein São Paulo SP Brazil Faculdade Israelita de Ciências da Saúde Albert Einstein, Hospital Israelita Albert Einstein, São Paulo, SP, Brazil.; 2 Universidade de São Paulo Faculdade de Medicina Clinical Immunology and Allergy Division São Paulo SP Brazil Clinical Immunology and Allergy Division, Faculdade de Medicina, Universidade de São Paulo, São Paulo, SP, Brazil.

**Keywords:** Teacher competencies, Faculty, Education, medical, Health education, Professional competence, Health personnel, School teachers

## Abstract

Teachers have the significant potential to facilitate alterations in the teaching-learning process that can positively affect student outcomes. In the healthcare field, exposure to effective teachers is associated with enhanced clinical performance and student knowledge. The literature explores several characteristics that increase the possibility of obtaining better outcomes when developed by teachers. These features are described from the perspectives of students, managers, and teachers. However, when considering teacher development programs, it is necessary to translate these characteristics, skills, and personality traits into competencies for training and enhancement. Therefore, in this review, we propose eight primary competencies that encompass the majority of these previously described characteristics: knowledge, communication skills, empathy, ability to facilitate engagement, preparation, organization, ability to assess learning progress, self-reflection, and continuous enhancement. All of these are trainable. However, teacher training is a complex and continuous process. Understanding these competencies is crucial for designing effective teacher development programs that offer measurable outcomes, reinforce teaching adherence, optimize enhancement timelines, and reduce institutional costs. This review discusses each of the eight competencies, their interrelationships, and their effects on healthcare education, offering a foundation for teachers and managers to facilitate educational excellence.

## INTRODUCTION

Education aims to empower individuals with knowledge and experience in a comprehensive, conscious, efficient, and effective manner.^(1)^ Education can be understood as a process that targets the development of competencies and skills in an individual.^(2)^

Teaching and learning are two facets of a single process guided by teachers.^(3)^ Teaching corresponds to actions, means, and conditions that facilitate instruction,^(4)^ aiming to provide students with the means to actively acquire knowledge.^(3,5)^ In contrast, learning is the process of acquiring knowledge and developing physical and mental skills that are organized and guided through teaching.^(6)^ Paulo Freire and other educators believe that teaching and learning are inseparable and that education is a dialogic process involving constant exchange. In this teaching-learning relationship, educators and students continually alternate their roles.^(7)^

High-quality teaching begins with effective planning, resulting in a coherent learning experience. Teachers should justify the significance and structure of the teaching content, their choice of pedagogical techniques, and how this combination addresses the learning needs of students, considering their prior knowledge, prejudices, and points of conceptual difficulty.^(8)^

In higher education, it is necessary to adapt the learning environment to adult learners by diagnosing their needs, formulating objective programs, and designing content that engages students in the learning process. The teacher plays the role of guiding the process, providing appropriate techniques and materials, and acting more as a catalyst than as an instructor. The teaching-learning experience is conducted through mutual responsibility between the students and teachers.^(9)^

In any context, three primary elements can be identified in the teaching-learning process: management or institutions, teachers, and students. The institution is responsible for ensuring that infrastructure, finances, strategic planning, people management, and pedagogical planning are proposed to professors and other topics involving students and public and private partner agencies are ensured.^(10)^ The student is the central focus of the entire teaching-learning process. In the past, students received knowledge from teachers, but currently, students play an active role in this process.^(11)^ In turn, the teacher is no longer a mere transmitter of information^(12)^ but assumes the role of leader or primary driver in the education process, aiming to teach students to interpret data and create from it^(1)^ – becoming a facilitator who supports students and identifies their individual learning needs.^(13)^

By analyzing this tripod (institutional, student, and teacher), it is possible to infer that innovations and initiatives aimed at enhancing the teaching-learning process can come from any of these groups. However, teachers may be the ones who best meet the strategic conditions that enable rapid and significant alterations in the educational scenario, such as student outcome.^(14-17)^ The primary reasons are as follows: teachers facilitate dialogue and alignment between students and managers; they can affect numerous individuals through their actions and reactions, creating a culture of high-quality education; and a single teacher committed to excellence and the well-being and learning of students generates a rebound effect, prompting students to expect similar standards from other teachers and management. Therefore, if teachers align their skills and drive their energy towards enhancing the teaching-learning process, these actions can propagate in institutions and spread to other professors and education managers.

Health education is a complex field that requires highly qualified specialists and professors – to train professionals with knowledge, problem-solving abilities, and self-directed learning skills – who can transfer these competencies to clinical practice.^(18)^ Like other sectors, healthcare education has undergone significant alterations in recent decades, with student-centeredness, integrated teaching, problem-based learning, novel assessment methods, rapid digitalization, and advances in data accessibility, technology, and science.^(18-21)^ Teachers play a crucial role in student development^(22)^ and are the primary contributors to academic performance. Evidence indicates that exposure to effective teachers is associated with enhanced clinical performance and greater student knowledge. Health educators are anticipated to teach and facilitate learning, planning, assessment, research, leadership, and educational management, while fostering a quality and safe educational environment.^(23)^ Redefining the role of teachers and their relationships with students is one of the primary actions driving the transformation of medical education, aiming to enhance the quality of services provided to users.^(7)^

Three primary categories of the characteristics of effective clinical professors were identified: 1) knowledge, skills, and abilities as physicians; 2) enthusiasm for medicine and teaching; and 3) positive human characteristics, such as communication skills and respect.^(24)^

Because teachers have the greatest potential to modify teaching and influence their students’ learning, it is crucial to understand the characteristics that enable them to positively contribute to the educational system. The literature offers various proposals describing the characteristics and attitudes of effective teachers, reflecting both students and teachers perspectives. We aimed to identify these characteristics and organize them to highlight areas for enhancement in teacher-development programs. Teacher development is undoubtedly one of the most powerful tools for enhancing the teaching-learning process. Additionally, recognizing what makes a teacher highly effective can contribute to designing programs that facilitate their development.

### Characteristics of an effective teacher: skills

Effective teachers are competent in helping students learn. Therefore, as observed in the literature, to achieve such effectiveness, teachers should acquire or develop specific competencies.^(25)^ These competencies are essential for student success in the teaching-learning process.^(26)^ The definition of competencies is not based on fixed, objective decisions but is dynamic, following advances in science and technology, with a constant goal of enhancing student outcomes.^(27)^

In any field, the fundamental qualities for teachers are knowledge and constant updates, investigative skills, cultural knowledge, acceptable training in pedagogy or andragogy, ethics, effective interpersonal skills,^(1)^ and verbal ability^(14)^ among others. Additionally, the inclination to change is a significant characteristic that helps teachers become more effective^(1)^ because it enables them to adapt to students’ needs.

Pedagogical competence – the ability to manage learning activities – is a crucial aspect of teaching that directly affects students’ learning. It involves the planning, implementation, and assessment of learning and the efforts of teachers to constantly enhance its quality.^(28)^

From the students’ perspective, an effective teacher should be friendly, patient, fair, consistent, approachable, communicative, calm, flexible, and interested in students’ concerns. Additionally, students emphasized that an effective teacher should demonstrate subject knowledge, pedagogical activity planning, use of diverse methodologies, support for learning, individualized student attention, respect for social and cultural diversity, continuous analysis and assessment of pedagogical processes, communication and cooperation in problem solving, research skills, enthusiasm for teaching and subject matter, and professional commitment.^(16,29-32)^

According to the teachers, in addition to knowledge, an effective teacher possesses verbal parsimony, humility, tolerance, fairness, patience, the ability to make decisions and control emotions, stability, adaptability, objectivity, humor, enthusiasm, cheerfulness, and affection.^(25,31)^ Educational practice requires emotional connection – teachers should build affective bonds with students because learning without such bonds lacks significance and results in gaps in the teaching-learning process.^(25)^ Teachers should effectively communicate and interact with their students, reward them equally for their behaviors, make classes enjoyable, demonstrate passion for teaching, provide accurate content, support critical thinking, manage classroom behavior, and assess students fairly – all of which contribute to students’ social and emotional development.^(31)^ Additionally, teacher preparation, organization, time management, achievement of learning objectives, clarity, the ability to challenge and support students, and timely feedback are essential qualities that affect learning.^(33)^

In the healthcare domain, competencies can be divided into three dimensions: knowledge, skills, and attitude. The knowledge dimension includes subject-specific knowledge, evidence-based teaching, ethical standards, and entrepreneurship. The skills dimension includes: 1) pedagogical abilities; 2) interactive and facilitative teaching; 3) assessment techniques; 4) problem-solving; 5) leadership; 6) research; 7) acquiring and conveying knowledge; 8) technological proficiency; 9) clinical expertise; 10) job-specific training; and 11) building relationships with students, including care and motivation. Their relationships with students incorporate characteristics, such as equality, honesty, encouragement of mutual respect, and appreciation. Teachers’ attitudes included positive attitude towards research, entrepreneurship, and personality traits (consistency, flexibility, admitting mistakes, and maintaining an open mind). ^(18)^

According to the Accreditation Council for Graduate Medical Education, effective healthcare educators should demonstrate competencies, such as medical knowledge, student-centeredness, interpersonal and communication skills, professionalism and role modelling, and practice-based reflections.^(34)^ Other characteristics associated with teachers’ efficiency include pedagogical knowledge, passion for teaching and students, ability to engage and simplify complex content, creativity, respect for students, experience, confidence, dedication to enhancing teaching and learning, practical ability, digital literacy, curriculum awareness, research experience, ethics, professionalism, and ability to integrate theory with practice.^(20,35)^

Teaching in a clinical setting requires both cognitive and non-cognitive skills, such as mastering the content, understanding the student-teacher relationship as an educational tool, engaging and motivating students, encouraging students to take responsibilities and make decisions when ready, and practicing self-reflection to be open to student feedback.^(36)^

**Figure 1:**
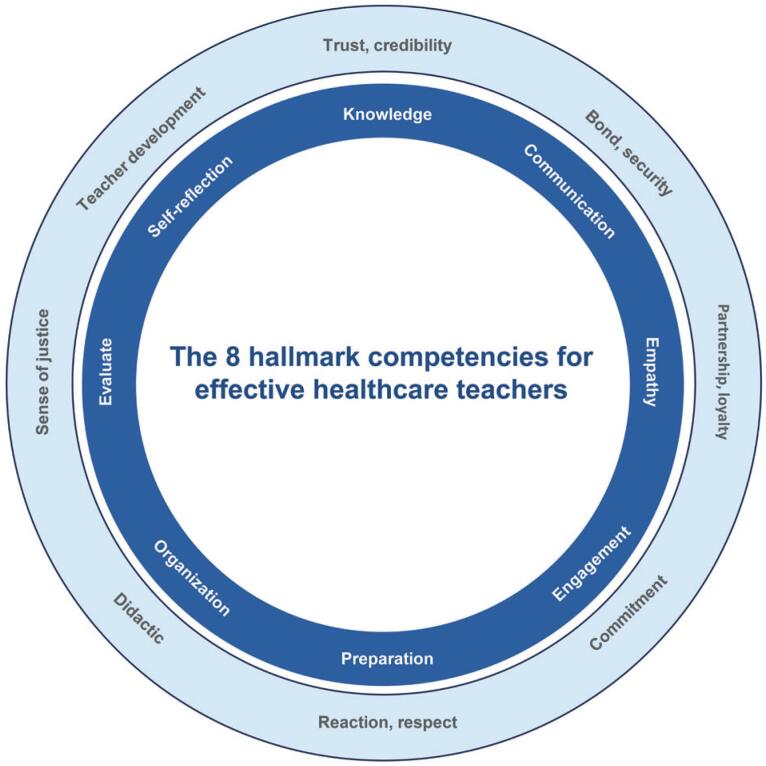
Eight characteristic competencies for effective teaching in the healthcare field

**Figure 2:**
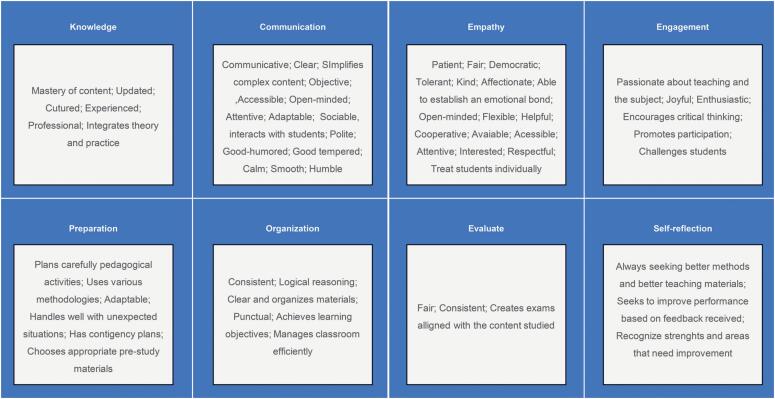
Terms used in literature associated with the characteristic competencies for effective teaching in the healthcare field

Based on reviews in the literature and considering the context of higher education in the healthcare field, we identified the primary characteristics and attitudes of effective teachers and grouped them into eight primary competencies: knowledge, communication skills, empathy, ability to facilitate engagement, preparation for classes, organization, ability to assess student learning, and self-reflection with continuous enhancement. Although other competencies are mentioned in the literature, we believe that they are directly or indirectly associated with these eight competencies. Similarly, personality-related characteristics or attitudes described in the literature intersect with our competencies (Table 1; Figures 1 and 2).

Competencies are cited in the dark blue ring, and their effects on the teaching-learning process are cited in the light blue ring.

**Table 1:** Characteristic competencies and the effect on teaching activity

Competence	Definition	Effect on teaching activity
Knowledge	Theoretical and practical	Credibility
	Technician	Trust
	Pedagogical and methodological	Engagement
	Technological	Security
	Professional and academic experience	
Communication skills	Transmit and receive data clearly, objectively, and in an appropriate voice tone and accessible language	Interpersonal relationship
		Security
	Adapt the message to the interaction and context	Engagement
	Speaking, listening, reading, and writing	Bond
	Verbal and nonverbal	Enables the other skills to be perceived by students
Empathy	Recognize and try to understand what the other is feeling, thinking, why they are acting and expressing themselves that way	Affective bond
		Complicity
		Partnership
	To manifest to other such recognition and understanding	Loyalty
		Safe learning environment
		Engagement
		Adaptability
		Meeting individual needs
Ability to promote engagement	Awaken the student's attention, curiosity, and interest in participating in the proposed activities without reward/grade, making them more motivated and committed to the learning process	Commitment and sharing of responsibility for learning with students
Preparation	Appropriate choice of study materials, teaching strategies, and classroom preparation, based on the learning objectives, target audience, resources, and available time	Security
		Good classroom control
		Reaction to any problems with contingency plans
		Tranquility
		Engagement
		Respect
Organization	Harmoniously conduct the class, presenting the content in a logical, linear, and coherent sequence	Students follow the teachers’ line of reasoning, and knowledge is exchanged in a harmonious way
		Support materials facilitate the understanding of content and are consistent with the speech and proposed activities
		Security
		Engagement
		Facilitation
Ability to assess the teaching- learning process	To assess the student's learning and progress in the acquisition of knowledge, skills, and attitudes, based on the learning objectives	Students feel respected
		Sense of justice
		Engagement
		Stimulus for study with the objective of learning (and not of passing the test)
		Learning tool (not only assessment)
Self-reflection and continuous enhancement	Think critically and constantly about their classes, activities, and other skills, reflecting on the students’ outcomes	Teacher engagement and commitment to contribute to better student outcomes
		Although students are not able to assess this process (internal of the teacher), they are affected by it

Subsequently, we offer a detailed analysis of each competency, highlighting common points and correlations among them.

### Knowledge

Teachers’ knowledge has been a subject of research for over 50 years and is recognized as fundamental to effective education.^(8)^ Notably, to accomplish their role, teachers should thoroughly understand their subject and the underlying scientific principles. Without this knowledge, teaching is impossible.^(1)^

This competence involves theoretical and technical knowledge and professional and academic experience in the subjects. Teacher's broad understanding of class subjects enables them to teach students the practical application of acquired knowledge. Additionally, this competence provides teachers and students with security and tranquility during the teaching-learning process. This approach offers credibility to the classes and minimizes improvisation and harmful errors.

For certain authors, this competence includes content and pedagogical knowledge necessary for teaching,^(18)^ knowledge of information technologies and ethical principles, and student needs. Teacher knowledge can be divided into academic, general pedagogical, and pedagogical content knowledge. Academic content knowledge can be defined as teachers’ factual knowledge of a specific topic. General pedagogical knowledge refers to the ability to implement general teaching skills in the classroom. Pedagogical content knowledge is based on theoretical knowledge and practical experience.^(8)^

All these aspects of knowledge are essential for effective teaching. Minor gaps in data and knowledge are common, specifically in dynamic and active classrooms. In these cases, other well-developed competencies can help to compensate for or complement these gaps. However, when teachers lack mastery of the subject or methodology, students notice it, thereby negatively affecting their engagement and learning.

### Communication skills

The literature identifies a combination of features to define effective teachers, with communication being a primary component.^(20,34,35,37-40)^ Communication involves conveying messages/knowledge in a clear, objective, and appropriate tone using accessible language. This involves listening, speaking, reading, and writing. Additionally, it can be defined as the ability to adapt the message to the context, respecting teacher-student interactions. Communication consists of two primary components: verbal (words) and nonverbal (attitudes, actions, appearance, posture, and use of classroom resources).^(41-43)^

The verbal component of communication includes speech intelligibility, speed, vocal intensity, and voice quality. The nonverbal components include teachers’ visual contact with students, gestures, movement in the classroom, sensitivity to class behavior, and moments of silence from students or teachers.^(44,45)^ Although verbal communication is easily observable, nonverbal communication is considered more effective because it consciously or unconsciously affects both teachers and students.^(45)^

Effective communication skills are crucial for public speaking, explaining content,^(20,34,35)^ and social skills aimed at students, other teachers, and administration. This enables the alignment of the expectations of all parties involved. Social skills involve expressing ideas, feelings, and opinions; maintaining or enhancing relationships with others; and resolving and managing social issues.^(46)^ Teacher-student interactions play a significant role in learning and teaching quality.^(47,48)^

Additionally, communication is crucial for maintaining relationships between teachers and students. Teachers who are accessible, open, and receptive tend to have better interpersonal relationships with their students, which can enhance student engagement and teaching-learning outcomes.^(49)^

The consequences of ineffective communication are illustrated through a few examples. Certain teachers, although experts in their fields, may struggle to adapt their content and approach to match students’ educational levels. This can result in overly complex explanations that affect students’ engagement and understanding of the subject. In another situation, ineffective communication creates a barrier between the teacher and students, making it difficult for students to ask questions. At times, miscommunication is disastrous because it suppresses psychological safety in the classroom, preventing students from expressing their needs or offering feedback – resulting in a toxic environment and chaotic learning process.

Therefore, communication is the competence that enables teachers to convey all other competences to their students. This significant skill enables teachers to share knowledge, express empathy, guide, and engage with their students.

### Empathy

Empathy has been defined in different ways in the literature and can be understood as the cognitive and affective ability to understand and respond to the emotional state of another person.^(50,51)^ It is the psychological ability to identify with others by putting oneself in their place and understanding their situation – what one is feeling, thinking, and why one is acting/thinking/expressing oneself in a specific manner.^(52-55)^

In the educational context, empathy involves assessing students’ needs, understanding their issues and learning barriers, enabling teachers to strengthen interactions with students and adapt education programs to meet their needs.^(34)^

Therefore, this competence is directly and closely associated with student-centered teaching that is possible only if the teacher combines empathy with other competencies, exercises friendly and helpful leadership with students, and attends to their needs.^(48)^ Empathy drives teachers to commit to both the academic success and well-being of their students.^(34)^ This approach enables teachers to recognize and address their students’ emotional needs by adjusting their teaching accordingly. Additionally, education plays a primary role in fostering close interpersonal relationships^(56)^ – specifically effective for teacher-student interactions – that trigger students’ cognitive, social, and professional development.^(57)^

Teachers’ empathy can reduce students’ stress levels, thereby positively affecting their engagement.^(58)^

Demonstrating to students that we understand their difficulties and concerns, that we have already been in their place, and supporting them throughout the process can contribute to better outcomes. However, this does not imply that the teachers are condescending. In contrast, it reduces the distance between teachers and students, thereby preventing communication barriers. Students who feel supported tend to be more engaged and confident in sharing doubts, providing feedback, and seeking help for academic and extra-academic issues that may affect their performance. Therefore, empathy contributes to developing safe learning environments.

### Ability to facilitate engagement

Student engagement is a primary component of success in higher education. ^(59)^ The ability to motivate and engage can be defined as a psychological process involving excitement, direction, intensity, and persistence of goal-directed voluntary actions.^(24)^

Teachers who are motivated and engaged in the teaching-learning process can enhance students’ motivation, which can positively affect their academic performance.^(24)^ One of the primary triggers of student engagement is their perception of teachers’ enthusiasm, passion for teaching, and the topics they discuss. Additionally, an effective teacher-student relationship is associated with greater engagement.^(60)^ Similarly, students’ perceptions of teachers as supporters of both academic and personal issues tended to increase their engagement.^(61)^

We understand that a teacher's ability to facilitate engagement lies in fostering students’ attention, curiosity, and interest in participating in the proposed activities without rewards/grades, thereby increasing their dedication, motivation, and commitment to the learning process.^(62, 63)^

Additionally, engagement encourages students and professors to participate in dialogue, provide feedback, and commit to effective courses. This enabled us to recognize the needs of all stakeholders, including students, teachers, administrators, patients, and the community.^(34)^ Moreover, it enables all parties involved to play their roles, thereby contributing to better care and learning outcomes.

Teachers who enjoy teaching, maintain a close relationship with students, are passionate about teaching, adapt content to the students’ learning level, effectively use resources in and out of the classroom, and understand the significance of their role tend to foster greater student engagement – a key to learning. It is useless for the teacher to master a topic, prepare for the class, develop activities with appropriate learning objectives, and avoid student involvement in the process.

### Preparation

The preparation for each class involves reflecting on the group of students, available resources and time, and specifically on class objectives in a way that transforms the teachers’ extensive knowledge and experience into concise, compatible data that students can effectively use during class. Additionally, this competence enables teachers to foresee possible challenges and prepare to overcome them, thereby ensuring effective outcomes.

Preparation for classes involves selecting reading materials for students, composing teaching strategies, preparing classrooms based on the learning objectives, target audience, available resources and time, and developing contingency plans for unexpected situations.^(64-66)^

Self-preparation and lesson planning are among the most effective teaching practices ^(67)^ that enhance teachers’ teaching experiences.^(68)^

An unprepared teacher, regardless of content mastery, may encounter classroom situations that hinder their ability to think creatively. They should get to know their students and have an effective idea of their prior knowledge to adapt their messages to their target audience. Additionally, they should identify any structural and technological issues that may arise to minimize unforeseen events or have alternative plans in case of such events. They should use previously scripted materials that are appropriate in size, language, and knowledge level to guide and ensure that students are at the same starting point. Moreover, they should prepare effective lesson plans with clear learning objectives. Finally, teachers should have sufficient information about situations that may affect their classes to minimize stressful situations that can affect student performance. Therefore, all these points should be considered in teachers prior preparation.

### Organization

Organization refers to the linearity with which the class unfolds, encompassing the presentation of audio-visual resources, time management, and the logical sequence of data.^(33)^ This is evident in the preparation material offered to students and the execution of the class, including its duration. Therefore, class literacy can be defined as the ability to conduct classes harmoniously while presenting the content in a logical, linear, and coherent sequence.

This competence is confused with preparation because the two are closely associated. However, it is easy to imagine a teacher who has knowledge of the content and has prepared for classes in a disorganized way, causing all their efforts and dedication to be wasted in class because of the lack of organization, linearity, and logic. This results in a sequence of activities that confuse students regarding concepts and processes.

Teachers’ organizations affect the entire teaching-learning process – before, during, and after class. This competence is becoming increasingly significant as digital platforms, applications, and artificial intelligence play major roles in student-teacher interactions.^(69)^

It should be present at all times of interaction, whether before, during, or after the class. Coherence must exist between these moments.

### Ability to assess the teaching-learning process

Because teaching and learning processes evolve in a course or discipline, various types of measurements and assessments should be performed to reveal whether students are learning and developing. Therefore, we can conclude that inaccurate and erroneous measurements do not reveal the true outcome of the process and may result in poor training. Therefore, the ability to assess student learning and progress in acquiring knowledge, skills, and attitudes is necessary for professors, students, and institutions to correct, enhance, or continue the currently adopted strategies. Additionally, correctly constructed and applied assessments engage and reassure students.^(20,34,35)^

If teachers do not use assessments to verify how the outcomes align with learning objectives, students may focus (consciously or unconsciously) on passing exams rather than integrating the knowledge and applying it.^(1)^ Therefore, this may negatively affect the outcomes of the teaching-learning process.

The increasing number of assessment tools has made it necessary for universities to include professors who specialize in assessing their faculties. Certain professors believe that assessment is the university's most significant responsibility and should receive more focus.^(11)^ Although assessment is undoubtedly one of the professor's crucial responsibilities, it becomes a learning tool when well designed. When students perceive self-compassion in this way, they exhibit greater engagement. In contrast, when students perceive a disconnect between what was taught/learned and the assessment requirements, they may feel a sense of injustice – that can discourage them and hinder the teaching-learning process.

### Self-reflection and continuous enhancement

Self-reflection corresponds to educators’ ability to think critically and constantly about their classes, activities, and students’ outcomes. ^(34)^ It has been recognized as essential for professional behavior and has therefore received growing attention in health education.^(70)^

This competence is associated with more persistent behavior and a search for better teaching methods. Therefore, teachers who practice constant self-reflection evolve faster and enhance their teaching skills, enabling them to meet the demands of teaching.^(24,71-75)^

This practice has already been used in teacher training to facilitate continuous professional development. Integrating this practice into teacher training enables teachers to assess their pedagogical performance, understand their strengths and limitations, and enhance their professional skills (self-knowledge, learning from experience, lifelong learning, and personal and professional growth). Therefore, reflective practice should be a part of continuous teacher development. Through this approach, teachers become autonomous learners^(72,73)^ and acquire a greater ability to identify and correct issues that affect their teaching performance.^(75,76)^

Teachers continually discover, incorporate, and apply novel skills and knowledge regarding student development and learning.^(25)^ Engaged and committed teachers always reflect on what worked and what did not in the classroom or outdoors to enhance their performance. Similarly, it is common to exchange experiences with other teachers, which facilitates reflection and the search for enhancement. This competence ensures that the teacher adapts to the context and alters teaching based on student needs, making it essential for enhanced teaching performance.

### Other competencies

Considering various competencies in the literature, the use of technology and emotional regulation share significant similarities that should be explored.

Teachers’ competence in information technology is crucial for enhancing the learning outcomes.^(77,78)^ With advances in the internet and software, teachers should update their skills and integrate such advances to engage digital-native students and automate processes – thereby enabling more time to support students and develop their skills.

Several authors consider this competence as an aspect of knowledge that is necessary for teachers to fully exercise their functions. Additionally, the selection and appropriate use of these resources are part of teachers’ preparation and organization.

Emotional management – a commonly cited competency – is part of the broader concept of emotional intelligence.^(79)^ Emotional intelligence is defined as the ability to perceive, access, generate, and regulate emotions to facilitate emotional and intellectual development.^(80)^ Individuals possess a set of skills that enable them to process their own emotions and those of others – using them to guide thoughts and behaviors for their benefit and that of others.^(81)^ This is essential for effective teaching.^(82,83)^ If teachers do not use emotional intelligence in their teaching, their expertise becomes less valuable, resulting in student failures.^(84,85)^ Additionally, emotional intelligence helps teachers deal with stress, thereby reducing their vulnerability.^(86,87)^ Teachers with high emotional intelligence are self-aware and capable of motivating themselves, thereby enhancing greater engagement.^(88-92)^ Additionally, they can self-reflect and assess their teaching strengths and weaknesses. Such teachers respect their students, accept them, recognize their emotions, consider their nonverbal behaviors to help them understand their feelings and concerns, and establish a healthy and productive relationship.^(93)^ Moreover, they plan an appropriate teaching environment using these emotions to enhance learning.^(91)^

Therefore, the competencies of communication^(94)^ and motivation/engagement^(88-92)^ encompass aspects of emotional intelligence, empathy, preparation, organization,^(79,91)^ the ability to assess learning,^(91)^ and self-assessment/continuous enhancement.^(91,95,96)^

## CONCLUSION

Teacher training is a complex and continuous process. Effective teachers always seek knowledge, novel techniques, and skills to help their students achieve effective outcomes. Additionally, effective teachers should always adapt to the needs and context of their students.

When developed by teachers, certain competencies increase the possibility of obtaining better outcomes. Therefore, identifying these competencies is fundamental when planning a teacher development program, because we believe that they can be developed and trained. In the healthcare field, faculty development programs are rarely structured around competencies. Although the competencies (knowledge and communication) are highly valued and widely discussed in the literature, no studies have assessed the effect of training other competencies in health education teachers. This can positively affect the teaching-learning process by enhancing the quality of teaching and resulting in better student outcomes. Additionally, we believe that prioritizing the development of these competencies can enhance the effective use of financial resources for teacher development.

Another crucial point from the institution's perspective is that recognizing these competencies enables more efficient recruitment by identifying them during the process, even before the hiring.

This recognition enables professors to self-assess and seek constant development and enhancement.
